# Lactate-Dehydrogenase 5 is overexpressed in non-small cell lung cancer and correlates with the expression of the transketolase-like protein 1

**DOI:** 10.1186/1746-1596-5-22

**Published:** 2010-04-12

**Authors:** Gian Kayser, Ahmad Kassem, Wulf Sienel, Luzie Schulte-Uentrop, Dominik Mattern, Konrad Aumann, Elmar Stickeler, Martin Werner, Bernward Passlick, Axel zur Hausen

**Affiliations:** 1 Institute of Pathology, University Hospital Freiburg, Freiburg, Germany; 2 Department of Thoracic Surgery, University Hospital Freiburg, Freiburg, Germany; 3 Department of Obstetrics and Gynecology, University Hospital Freiburg, Freiburg, Germany

## Abstract

**Aims:**

As one of the five Lactate dehydrogenase (LDH) isoenzymes, LDH5 has the highest efficiency to catalyze pyruvate transformation to lactate. LDH5 overexpression in cancer cells induces an upregulated glycolytic metabolism and reduced dependence on the presence of oxygen. Here we analyzed LDH5 protein expression in a well characterized large cohort of primary lung cancers in correlation to clinico-pathological data and its possible impact on patient survival.

**Methods:**

Primary lung cancers (n = 269) and non neoplastic lung tissue (n = 35) were tested for LDH5 expression by immunohistochemistry using a polyclonal LDH5 antibody (ab53010). The results of LDH5 expression were correlated to clinico-pathological data as well as to patient's survival. In addition, the results of the previously tested Transketolase like 1 protein (TKTL1) expression were correlated to LDH5 expression.

**Results:**

89.5% (n = 238) of NSCLC revealed LDH5 expression whereas LDH5 expression was not detected in non neoplastic lung tissues (n = 34) (p < 0.0001). LDH5 overexpression was associated with histological type (adenocarcinoma = 57%, squamous cell carcinoma = 45%, large cell carcinoma = 46%, p = 0.006). No significant correlation could be detected with regard to TNM-stage, grading or survival. A two sided correlation between the expression of TKTL1 and LDH5 could be shown (p = 0.002) within the overall cohort as well as for each grading and pN group. A significant correlation between LDH5 and TKTL1 within each histologic tumortype could not be revealed.

**Conclusions:**

LDH5 is overexpressed in NSCLC and could hence serve as an additional marker for malignancy. Furthermore, LDH5 correlates positively with the prognostic marker TKTL1. Our results confirm a close link between the two metabolic enzymes and indicate an alteration in the glucose metabolism in the process of malignant transformation.

## Introduction

As one of the five lactate dehydrogenase (LDH) isoenzymes, LDH5 has the highest efficiency to catalyze pyruvate transformation to lactate. LDH5 overexpression in cancer cells induces an upregulated glycolytic metabolism and reduced dependence on the presence of oxygen. According to Warburg, malignant tumors generate lactate even in the presence of sufficient oxygen supply (Warburg effect) [1]. Upon this Thompson postulated a shift from oxidative phosphorylation to anaerobic glycolysis as a key element for malignant transformation of cells [2]. This change in the cells' glucose metabolism is not due to mitochondrial defects but results in a higher capacity and efficiency to facilitate cellular growth, thus to synthesize fatty acids, amino acids and reductive equivalents necessary for nucleic acid synthesis. The generation and consumption of oxaloacetat - kata- and anaplerosis - within the citric acid cycle (TCA) is supposed to play a key role in this phenomenon as intermediates of the TCA are needed for fatty acid synthesis which in turn are necessary for cell membrane composition [3,4]. Pyruvate as a precursor of acetyl-CoA is therefore a pivotal substrate in tumor cell glycolysis [3,4].

Nucleic acid synthesis is initiated via the pentose phosphate pathway (PPP) which also uses glucose as source molecule.

Recent studies on glucose degradation pathways of malignant tumors also postulate a shunt between the PPP and the aerobic glycolysis (Embden-Meyerhof-pathway; EMP) with Glycerinaldehyde-3-phosphate (G3P) possibly representing the linking molecule between the two. According to these results G3P is generated from xylose-5-phosphate (X5P), one endproduct of the PPP by transketolase like protein 1 (TKTL1). Therefore, TKTL1 could be one of the key enzymes in malignant cell glucose metabolism [5]. In a recent study we were able to demonstrate that overexpression of TKTL1 is associated with a poor outcome in patients with non small cell lung cancer (NSCLC).

Since TKTL1 is supposed to be a key molecule between the EMP and the PPP TKTL1 overexpression would result in high intracellular G3P and pyruvate levels. The latter would then be either transferred into the TCA or be catalyzed to lactate by LDH5. In malignant tumors a fair amount of lactate is produced most probably by LDH5 out of pyruvate [3].

Therefore, we investigated LDH5 expression in non-small cell lung cancer (NSCLC), its correlation with TKTL1 and its impact on clinico-pathological parameters such as tumor stage and survival in a large and well characterized cohort of NSCLC.

## Materials and methods

After approval by the ethical committee (No.: 14/04) of the University of Freiburg and after written informed consent, 269 patients suffering from non-small cell lung cancer (NSCLC) were included in this study. Operation specimens, removed with curative intent between January 1^st ^1989 and December 31^st ^2004, were obtained from the Department of Thoracic Surgery, University Hospital Freiburg. Pathologic diagnosis and staging was performed at the Institute of Pathology, University Hospital Freiburg according to the UICC TNM-system, 6^th ^edition [6]. All specimens were fixed routinely in 4% buffered formaline for 24 to 48 h and subsequently paraffin embedded. Reevaluation of the histological diagnosis was performed by three independent experienced pathologists (G.K., A. z. H. and D. M.).

Tissue microarrays (TMA) of the 269 primary NSCLC were generated by taking three cores from each tumor with respect to tumor heterogeneity. A control set of 34 non-neoplastic lung tissues containing alveolar as well as bronchial areas was also established from tumorfree tissue sample within the same cohort.

The clinico-pathological data of these 269 patients are summarized in table 1.

**Table 1:** Summary of the clinico-pathologic data of 269 lung cancer patients

Age	35 - 83 years	63.47 +/- 9.53 years
	**Number**	**Percent**

**sex**		

Male	191	71.0

Female	78	29.0

		

**Histologic entity**		

Adenocarcinoma (AC)	90	33.5

Squamous cell carcinoma (SCC)	106	39.4

Large cell carcinoma (LC)	73	27.1

		

**pT-category**		

Not assessable	2	0.7

pT1	71	26.4

pT2	147	54.6

pT3	26	9.7

pT4	23	8.6

		

**pN-category**		

pNx	9	3.3

pN0	147	54.6

pN1	49	18.2

pN2	46	23.0

pN3	2	0.7

		

**UICC**		

Not assessable	6	2.2

1A	50	18.6

1B	75	27.9

2A	8	3.0

2B	37	13.8

3A	63	23.4

3B	23	8.6

4	7	2.6

### Immunohistochemical staining

**Figure 1:**
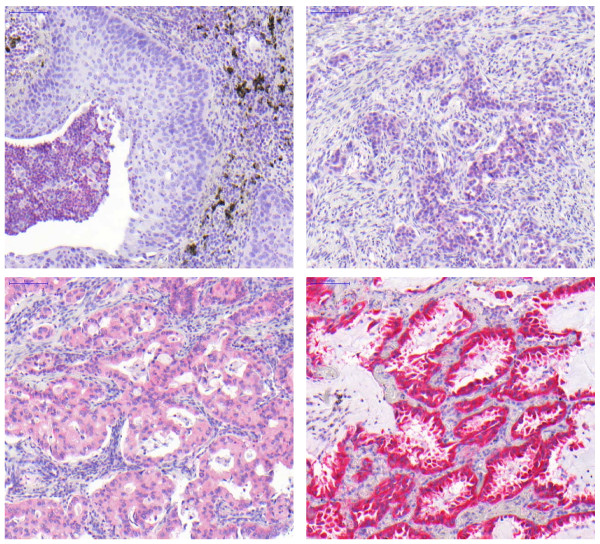
**Immunohistochemical staining of LDH5 in NSCLC**. Upper left: Squamous cell carcinoma without immunohistochemically detectable expression of LDH5; Upper right: Adenocarcinoma with weak expression of LDH5 - score 1; lower left: Adenocarcinoma with moderate expression of LDH5 - score 2; lower right: Adenocarcinoma with strong expression of LDH5 - score 3. (200×)

Three μm thick paraffin sections were taken from all TMAs and used for immunohistochemistry. After dewaxing the sections were incubated with TRS (Target retrieval solution, Dako) for 20 minutes at pH 9 and afterwards with proteinase K for 5 minutes. The endogenous avidin-biotin was blocked by the commercial biotin blocking system (DAKO) for 10 minutes. Subsequently, the slides were washed in Tris/saline buffer (TBS) twice and incubated with 1% goat serum for 30 minutes to block unspecific binding sites. Polyclonal rabbit anti-LDH5 antibody (ab53010) was applied for 30 min at room temperature. After rinsing with TBS the slides were incubated with biotinylated anti-rabbit immunoglobulins for 15 minutes at room temperature and treated with streptavidin-peroxidase (DAKO) for 10 minutes. Staining was performed by using 3-amino-9-ethylcarbazole (AEC) substrate and counterstained with hematoxylin (figure 1).

The following evaluation-scoring system was applied: 0 - "negative"; 1 - "weak staining"; 2 - "moderate staining"; 3 - "strong staining". Furthermore the percentage of positive tumor cells was calculated: For each TMA-core all malignant cells with nuclear and/or cytoplasmic positivity for LDH5 were considered in relation to the absolute number of tumor cells (figure 1).

Upon analysis three cases had to be excluded from the study as no tumor tissue was present on the immunohistochemically stained sections.

Of 201 tumor samples data for TKTL1 staining was available.

### Statistical analyses

Statistical analysis regarding the correlation of LDH5 expression with clinico-pathological parameters as well as with the expression of TKTL1 was performed using the Number Cruncher Statistical System Software release 2007 (NCSS 2007) as well as the SPSS software version 15 for windows for reevaluation of the statistical results. The analyses included a Pearson-correlation, as well as chi-square test, Student's t-test, one-way ANOVA, Wilcoxon Signed Rank test, Kolmogorov-Smirnov test, log-rank test for Kaplan-Meier survival analysis and multivariate Cox regression analysis.

## Results

### LDH5 is overexpressed in malignant neoplastic lung tissue

In none of the 34 non-neoplastic lung tissue samples LDH5 expression could be detected by immunohistochemistry. In contrast, only 28 cases (10.53%) of all the NSCLC samples were negative for LDH5 expression. Approximately 90% of all NSCLC revealed LDH5 expression. The mean percentage of positive tumor cells within our collection was 49.44% (+/- 28.62; median: 56.67%) with a mean staining intensity of 1.28 (+/- 0.81; median: 1.33). Thus, LDH5 is overexpressed in NSCLC with high statistical significance compared to non-neoplastic lung tissue (p < 0,001).

### LDH5 shows stronger expression in differentiated NSCLC, especially adenocarinomas

Analysis of the three different main entities of non-small cell lung carcinomas revealed a mean staining intensity of 1.63 +/- 0.86 for adenocarcinomas (AC), 1.12 +/- 0.74 for squamous cell carcinomas (SCC) and 1.09 +/- 0.81 for large cell carcinomas (LC) with a mean percentage of positive tumor cells of 57.35% +/- 28.74 for AC, 44.97% +/- 27.47 for SCC and 46.35% +/- 28.48 for LC. In a one sided ANOVA analysis AC revealed a statistically significant higher expression than SCC or LC (intensity: p = 0.006; percentage: p < 0.001). Combining the prognostic favorable ACs with SCCs and comparing their expression with that of LCs we observed a decrease in the percentage of positive tumor cells from 50.61% +/- 28.66 for ACs and SCCs to 46.35% +/- 28.48 for LCs (p = 0.279) and a statistically significant decrease in staining intensity from 1.35 +/- .83 for ACs and SCCs to 1.09 +/- 0.70 for LCs (p = 0.018). No correlation of LDH5 expression could be detected for histopathologic grading.

### LDH5 expression is correlated with mediastinal lymphnode metastases and smoking

No statistically significant correlation between LDH5 expression and the individual pT or pN stage could be detected. Tumors having spread to regional lymphnodes showed no different LDH5 expression pattern than those without lymphnode metastases. Comparing the tumors with no or positive hilar lymphnode metastases (pN0 and pN1) with those having already spread to mediastinal lymphnodes (pN2 and pN3) a statistical trend (percentage of positive cells) and a statistical significance (staining intensity) for an increase of LDH5 expression was found (table 2).

**Table 2:** LDH5 expression in correlation with lymphonodal metastasis revealed a statistically significant difference in regard to the intensity score between no (pN0) and hilar lymphnode metastases (pN1) and mediastinal lymphnode metastases (pN2 and pN3).

	pN0 and pN1	pN2 and pN3	p-value
Percentage of positive tumors cells	47.12 +/- 29,.3	55.62 +/- 26.33	0.068

Staining intensity	1.22 +/- 0.82	1.43 +/- 0.75	0.036*

* statistically significant (p < 0.05)

In patients currently smoking or having smoked once expression of LDH5 measured by staining intensity was decreased as compared to non-smokers (1.57 +/- 0.83 in non-smokers vs. 1.24 +/- 0.80 in smokers; p = 0.030; t-Test).

### LDH5 expression correlates positively with TKTL1 expression

In a linear regression analysis, a positive correlation between the percentage of positive tumor cells for LDH5 and TKTL1 was found statistically significant (R-squared = 0.0233; p = 0.0317) meaning a simultaneous increase in the percentage of positive tumor cells within our cohort for TKTL1 and LDH5 expression. For the intensity of staining no significant correlation between the expression of the two enzymes could be detected.

### Overall survival analysis

**Figure 2:**
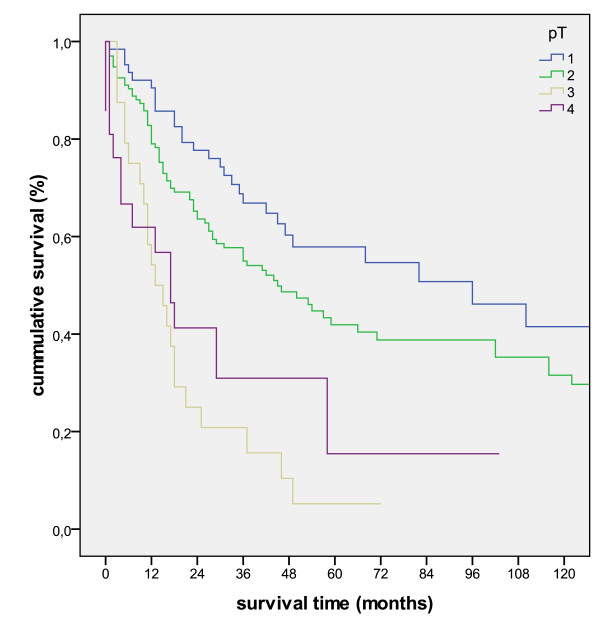
**Kaplan-Meier survival curve for the separate pT-stages (p < 0.001)**.

**Figure 3:**
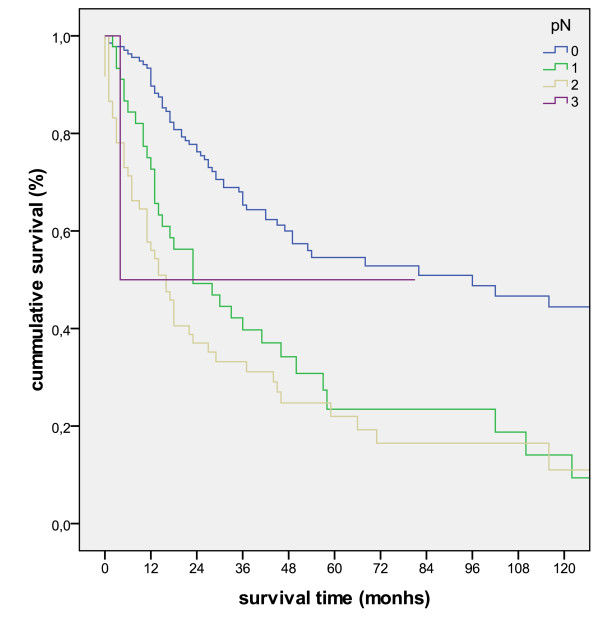
**Kaplan-Meier survival curve for the separate pN-stages (p < 0.001)**.

**Figure 4:**
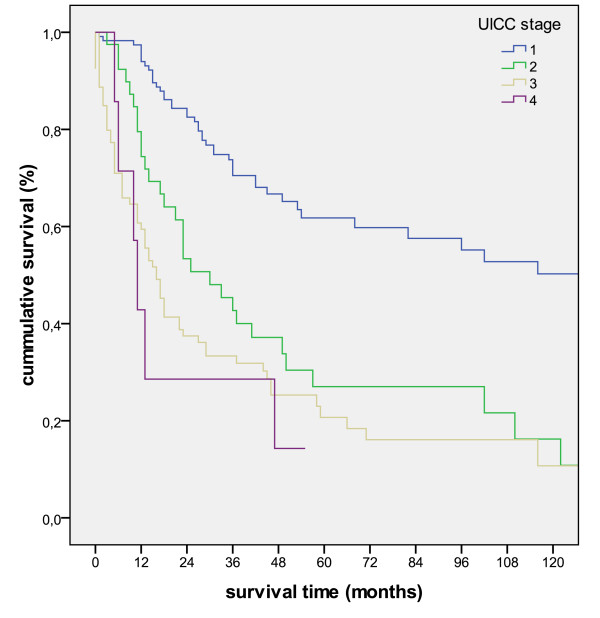
**Kaplan-Meier survival curve for the separate UICC-stages (p < 0.001)**.

The Kaplan-Meier survival analyses for the pT, pN and UICC-stage is shown in figure 2, figure 3 and figure 4.

**Figure 5:**
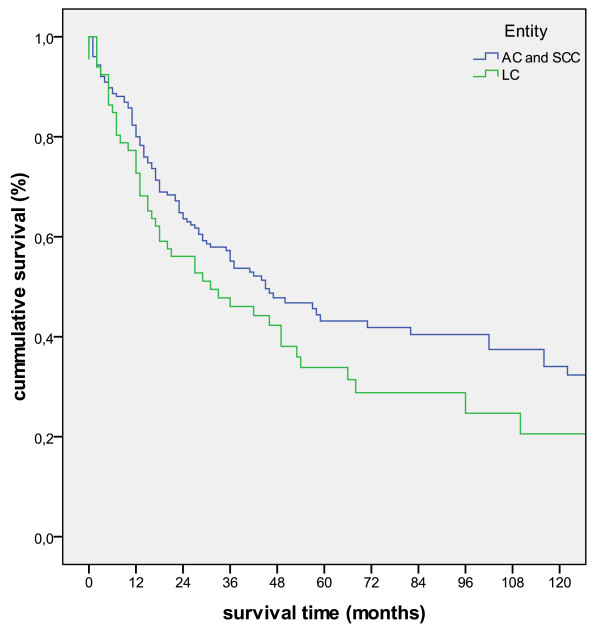
**Kaplan-Meier survival curve in regard to histologic type (p = 0.123)**.

In our patient cohort ACs and SCCs had a slightly better outcome as compared to LCs (p = 0.28 (Log-Rank)). Therefore we grouped the two "differentiated" carcinoma entities into one group which then showed a tendency for a better survival in comparison to LCs (p = 0.123; figure 5).

**Figure 6:**
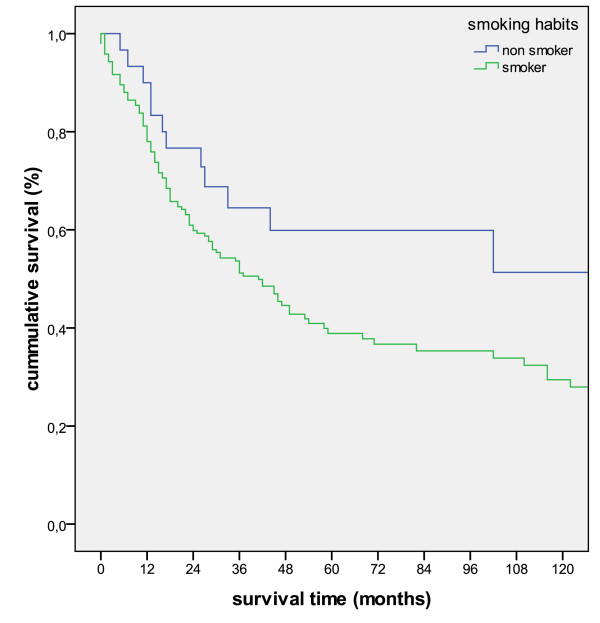
**Kaplan-Meier survival curve comparing smokers vs non-smokers (p = 0.087)**.

Concerning smoking habits non-smokers had a statistically significant better outcome than smokers (p = 0.047, figure 6).

### LDH5 overexpression favors a better survival in non-smokers

**Figure 7:**
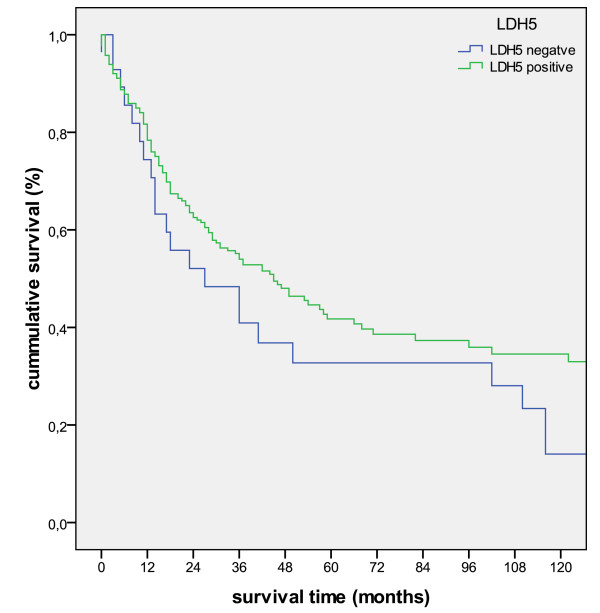
**Kaplan-Meier survival curve for LDH5-positive vs LDH5-negative tumors (p = 0.165)**.

Survival analysis comparing LDH5-negative with LDH5-expressing tumors did not reveal statistical significance (p = 0.165; figure 7). When defining a cut-off of 50% positive tumor cells (mean percentage of positive cells 49.44% +/- 28.62) as LDH5 overexpression no difference in survival was detected, either (p = 0.713). Subdividing the cohort according to clinico-pathologic parameters into different prognostically relevant groups (nodal status, UICC-stages) survival analysis in regard to overexpression of LDH5 did not reach statistical significance.

**Figure 8:**
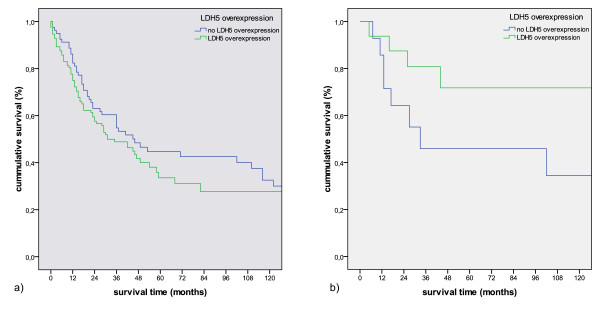
**Kaplan-Meier survival curve comparing overexpression of LDH5 in smokers (a) (p = 0.16) and non.smokers (b) (p = 0.098)**.

In contrast to this, LDH5 overexpression was in favor of a better prognosis in non-smokers (p = 0.09). This favorable effect was not observed in the smokers' group (figure 8).

## Discussion

Malignant tumors inhabit a complex carbohydrate metabolism which differs from that of non-neoplastic cells with two main paradigms: 1) malignant cells produce large amounts of lactate even in the presence of sufficient oxygen for aerobic glycolysis [1] 2) intermediates of the TCA are used for fatty, amino and nucleic acid synthesis [3,4]. Thus, the extensive glucose uptake of cancer cells is needed not only for energy supply but also to provide the components for cellular growth and a high amount of reducing equivalents such as NADPH [3,4]. To accomplish this, high levels of pyruvate are needed which can be introduced either into the TCA, converted into Acetyl-CoA or degraded to lactate by LDH [4,5]. The latter results in an excess of lactate [3,4]. In concordance with this theory of changes in tumor cell metabolism Koukourakis et al. showed that LDH5 was overexpressed in 36 out of 76 non-small lung cell cancers (47%) [7]. In our cohort none of the 34 non-neoplastic tissue controls was positive for LDH5, while 89.43% of tumors were positive for LDH5. As this is highly statistically significant (p < 0.001) LDH5 can also be proposed as an immunohistochemical marker for neoplasias in the lung.

LDH is composed of two different subunits, LDH-H and LDH-M. Each LDH isoenzyme is composed of 4 subunits, thus recombination of these subunits leads to 5 different isoenzymes, LDH1 with 4 LDH-H subunits to LDH5 with 4 LDH-M subunits. These isoenzymes differ in their specific substrate binding which is highest for lactate in LDH1 and for pyruvate in LDH5, respectively [8]. Maekawa et al. recently reported that the promoter for the LDHB gene is silenced in cancer cells via hypermethylation. This leads to restricted transcription of the LDH-M subunit eliminating transcription of the isoenzyms LDH1 to LDH4 [9,10]. Upon this pathogenetical background our results with LDH5 being overexpressed in NSCLC are in agreement with the proposed tumor biology.

We observed decreasing LDH5 expression levels in differentiated carcinomas (SCC and AC) compared to LCs. No correlation between these two subgroups concerning the percentage of positive cancer cells could be detected. This suggests that the decrease in staining intensity could be due to the larger cellular volume in LCs in terms of a "dilution effect" for staining intensity.

Our cohort of lung cancers showed comparable data in terms of patients' age, disease stage and survival as published in the literature [11-14]. As the patients in our cohort received primary surgery for curative treatment, small cell lung carcinomas (SCLC) were not included into the study due to the small number of available surgical specimens. At first glance, the frequency of large cell carcinomas is relatively high (27.1%). But taking into account that published data with proportions of around 8 to 10% for LCs usually include SCLC and rare entities [12], adjusting these figures in analogy to our cohort by recalculating the percentages without SCLC gives proportions of around 22% for large cell carcinomas [12]. This is in the range of our cohort.

**Figure 9:**
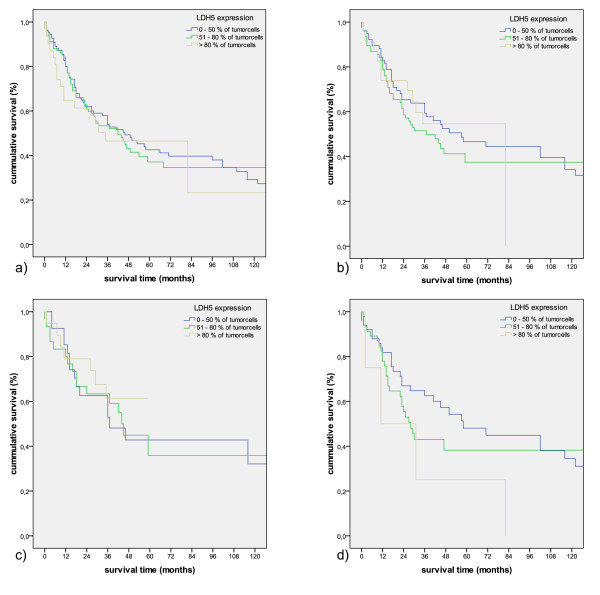
**Kaplan-Meier survival curves comparing LDH5 expression according to Koukourakis et al **[13]**in all NSCLC (p = 0.785) (a), AC combined with SCC (p = 0.551) (b), AC (p = 0.662) (c) and SCC (p = 0.145) (d)**.

Koukourakis et al. reported a link between LHD5 overexpression and survival in a set of 76 SCCs and 36 ACs [15]. Taking the same parameters we could not find similar significant associations, even by omitting LCs and only investigating ACs and SCCs (figure 9; p = 0.785 for all NSCLC, p = 0.551 for AC and SCC combined, p = 0.662 for AC, p = 0.145 for SCC). The diversity between the two studies can be explained not only by the fact that we used a different antibody but also by the investigated figures: the median percentage of stained tumor cells was calculated by 56.67% in our cohort, Koukourakis experienced a median of 80% of cytoplasmatic positive carcinoma cells. Thus, we were not able to reproduce the cited results in a large and well characterized cohort of NSCLC patients.

In order to further analyze the impact of LDH5 expression on patient's survival a positive correlation only with advanced (mediastinal) lymphnode metastases (pN0 + pN1 vs pN2 + pN3) and a positive correlation between the expression of LDH5 and TKTL1 was apparent. The latter is overexpressed in a large portion of NSCLC [16] and is an independent indicator for aggressive subtypes in NSCLC, as we have shown in another study. It could therefore be discussed if LDH5 overexpression does not appear as a linear function with a steady increase from little to highly aggressive cancers but rather in smaller steps which then reach a saturation level. Fantin et al. describe the necessity for upregulation of LDH in cancer cells for malignant transformation. Their results also stress the close link between lactate production and oxidative phosphorylation. This link is according to their results dependent on the upregulation of LDH but also regulated through metabolite concentrations [17]. This also supports our data and hypotheses.

The metabolism of healthy and functionally active cells is optimized for productivity in terms of fulfilling their duty to synthesize, degrade, transport or contract. In contrast to this, malignant tumor cells do not meet these demands but only strive for cellular growth and mitosis. According to this theory high amounts of ATP as demanded within healthy cells are not only not required but act contra-productive for the synthesis of basic elements for cellular growth such as nucleic acids, proteins and fatty acids. By degradation of pyruvate to lactate the pool of reductive equivalents on the one hand and the availability of citric-acid cycle intermediates for fatty and amino acid synthesis on the other hand is raised [3,4]. Our results which describe the overexpression of LDH5 in tumor cells and its correlation with TKTL1 further supports this theory of a glucose metabolism optimized for cellular growth within malignant tumors.

## Conclusion

The results of our study indicate that immunohistochemical analysis for LDH5 expression in NSCLC is highly specific to differentiate malignant neoplasia from healthy lung tissue. Furthermore, although expression of LDH5 did not directly correlate with patient's survival in our cohort, significant differences of its expression in correlation with the most indicative prognostic parameter - the pN stage - was present. This could indicate that the level of LDH5 expression is of prognostic value in NSCLC. In this context we could also demonstrate that LDH5 expression was positively correlated with the expression of TKTL1, which does not only inhabit a close functional link to LDH5 in the glucose metabolism of malignant tumors but is also an indicator for aggressive tumor biology. As LDH5 plays a pivotal role in tumor glucose metabolism our data may open new perspectives for novel chemotherapeutic strategies in NSCLC.

## Competing interests

The authors declare that they have no competing interests.

## Authors' contributions

GK participated in the study design, set up the immunohistochemical scoring system, performed the statistics and drafted the manuscript. AK performed the immunohistochemical stainings and their scoring. WS was involved in characterizing the cohort and performed parts of the statistics section as well as drafting the manuscript. LS-U updated the survival data of the cohort and enlarged number of included patients. DM and KA were involved in immunohistochemical stainings, and in proof readings of the manuscript. ES was involved in proof readings of the statistical results. MW and BP participated in the study design and its coordination. AZH conceived the study and drafted the manuscript. All authors read and approved the final manuscript.
